# Effects of testosterone treatment on hypothalamic neuroplasticity in female-to-male transgender individuals

**DOI:** 10.1007/s00429-017-1494-z

**Published:** 2017-08-17

**Authors:** Georg S. Kranz, Andreas Hahn, Ulrike Kaufmann, Martin Tik, Sebastian Ganger, René Seiger, Allan Hummer, Christian Windischberger, Siegfried Kasper, Rupert Lanzenberger

**Affiliations:** 1 Neuroimaging Labs (NIL) PET, MRI, EEG and Chemical Lab, Department of Psychiatry and Psychotherapy, Medical University of Vienna, Waehringer Guertel 18-20, 1090 Vienna, Austria; 2 Department of Obstetrics and Gynecology, Medical University of Vienna, Vienna, Austria; 3 MR Centre of Excellence, Center for Medical Physics and Biomedical Engineering, Medical University of Vienna, Vienna, Austria

**Keywords:** Hypothalamus, Testosterone, Female-to-male, Transgender, Diffusion-weighted imaging

## Abstract

Diffusion-weighted imaging (DWI) is used to measure gray matter tissue density and white matter fiber organization/directionality. Recent studies show that DWI also allows for assessing neuroplastic adaptations in the human hypothalamus. To this end, we investigated a potential influence of testosterone replacement therapy on hypothalamic microstructure in female-to-male (FtM) transgender individuals. 25 FtMs were measured at baseline, 4 weeks, and 4 months past treatment start and compared to 25 female and male controls. Our results show androgenization-related reductions in mean diffusivity in the lateral hypothalamus. Significant reductions were observed unilaterally after 1 month and bilaterally after 4 months of testosterone treatment. Moreover, treatment induced increases in free androgen index and bioavailable testosterone were significantly associated with the magnitude of reductions in mean diffusivity. These findings imply microstructural plasticity and potentially related changes in neural activity by testosterone in the adult human hypothalamus and suggest that testosterone replacement therapy in FtMs changes hypothalamic microstructure towards male proportions.

## Introduction

As control center of the neuroendocrine system, the hypothalamus is the main locus of action of sex hormones in triggering sexual motivation, cognition, and behavior. During perinatal brain development, sex hormones determine the adult hypothalamic neural circuitry into a male pattern or a female pattern (Arnold 2009). Moreover, there is growing appreciation that sex hormones also shape hypothalamic and other subcortical brain structures in adolescence (McCarthy et al. 2009; Campbell and Herbison 2014; Ahmed et al. 2008). In adulthood, these “organizational” effects are then replaced by “activational”, plastic and reversible actions of sex hormones. These include not only feedback actions on gonadotropin releasing hormone (GnRH) neuron firing and GnRH release (Mizuno and Terasawa 2005; Kenealy et al. 2013), but also influence ventromedial nucleus neuron firing involved in the control of glucose and lipid metabolism (Xu et al. 2011), as well as preoptic neuron firing involved in the regulation of body temperature (Silva and Boulant 1986). Estrogen reversibly modulates dendritic spine density, length, and terminal branch number in the ventromedial nucleus of the rat (Madeira et al. 2001). Structural neuroimaging in humans further indicates state-dependent changes in hypothalamus volume across the menstrual cycle (Tu et al. 2013).

Hypothalamic microstructure and function can be investigated using diffusion-weighted imaging (DWI) in humans. In one study, authors observed increased diffusion values in obese compared to non-obese participants in hypothalamus and other subcortical regions (Alkan et al. 2008). Another study compared hypothalamic diffusion parameters in fasted versus fed individuals. Mice as well as humans showed decreased diffusion in the fasted state, which may suggest a cellular swelling response associated with hypothalamic activation (Lizarbe et al. 2013). Indeed, recent DWI investigations indicate a potential link between water diffusion changes and neural activation via accompanying cell swelling (Le Bihan and Iima 2015; Le Bihan et al. 2006). Furthermore, diffusion changes have been associated with astrocyte swelling and used to study short-term gray matter adaptations after learning in the hippocampus (Sagi et al. 2012). However, to our knowledge, only one study so far has probed DWI to quantify sex hormone-induced neuroplastic adaptations in the hypothalamus in humans. Baroncini et al. (2010) investigated the effects of an artificial menstrual cycle on hypothalamic microstructure and observed decreased water diffusion upon inhibition of HPG-axis. Therefore, we aimed to further investigate a potential influence of sex hormones on hypothalamic microstructure using DWI.

The investigation of female-to-male transgender individuals (FtMs) seeking for sex hormone replacement therapy provided us the unique opportunity to accomplish this endeavor in a causal way. Hormone replacement therapy in FtMs consists mainly of high dosages of testosterone to adjust the physical appearance to the desired gender. Masculinization including the induction of body and facial hair growth and lowering of voice pitch is evident within 4 months and continues to develop beyond 1 year (Levy et al. 2003). Therefore, we explored the possibility that hypothalamic water diffusion would significantly change within 4 months of hormone intake.

## Materials and methods

### Subjects

A total sample of *n* = 50, consisting of 25 FtMs and 25 controls (12 FCs and 13 MCs), were included and analyzed. Data from these participants were taken from a larger study and have partly been published previously (Hahn et al. 2016; Kranz et al. 2014; Spies et al. 2016). Subjects’ age was comparable between groups (FtM 27.24 ± 6.2, FC 24.42 ± 5.4, MC 28.77 ± 6.5, mean ± SD, *p* = 0.21, ANOVA). FtMs were diagnosed using DSM-IV and ICD-10 in several semi-structured, sociodemographic, clinical, and psychiatric interviews. They were recruited from the transgender outpatient unit of the Department of Obstetrics and Gynecology, Medical University of Vienna, were naïve to sex hormone treatment, and wanted sex reassignment. All participants underwent standard medical examination, electrocardiogram, routine laboratory tests, and the Structured Clinical Interview for Diagnostic and Statistical Manual of Mental Disorders, Fourth Edition (DSM-IV-TR) to rule out physical, psychiatric, and neurological disorders (except for gender dysphoria in FtMs). All participants received financial compensation for their participation. After complete description of the study to the participants, written informed consent was obtained. The study was approved by the Ethics Committee of the Medical University of Vienna. All procedures were in accordance with the ethical standards of the institutional and/or national research committee and with the 1964 Helsinki declaration and its later amendments or comparable ethical standards.

### Study design and treatment protocol

The study was designed as a longitudinal monocenter study. FtMs underwent a baseline scan before start of hormone treatment (MRI 1), a second scan 1 month after treatment start (MRI 2) and a third scan 4 months after treatment start (MRI 3). Hormone treatment followed protocols routinely implemented at the Department of Obstetrics and Gynecology, Unit for Gender Dysphoria, at the Medical University of Vienna. 22 FtMs received 1000 mg testosterone undecanoate every 12 weeks (Nebido 250 mg/mL, 4 mL vial, intramuscular), whereas 3 FtMs received 50 mg testosterone transdermally (Testogel 5 mg-bag per day). In addition, one case further received 10–15 mg lynestrenol (Orgametril 5 mg, oral) daily. Female and male controls received no treatment but were measured three times using the same time schedule as given above.

### Blood sampling

Blood sampling was done shortly before MRI scanning around midday (i.e., approximately 4 h after waking). The analysis of plasma levels of testosterone (*T*), bioavailable testosterone (bioav *T*), estradiol (*E*), and sex hormone-binding globulin (SHBG) was done by the Department of Laboratory Medicine, Medical University of Vienna (http://www.kimcl.at). The free androgen index (FAI) was computed as FAI = testosterone/SHBG × 100.

### Magnetic resonance imaging (MRI)

Participants underwent a 4.56 min whole-brain diffusion-weighted image (DWI) scan on a 3 T TIM Trio Scanner (Siemens) using a 32-channel head coil. DWI was acquired with a single-shot diffusion-weighted echo planar imaging sequence (TE, 83 ms; TR, 8700 ms; flip angle, 90°; image resolution, 1.64 mm isotropic; *b* value, 800 s/mm^2^; 70 axial slices) in 30 diffusion encoding directions and one non-diffusion-weighted reference image (*b* = 0). Polyurethane cushions placed between the head coil and subjects’ heads minimized head movement.

In addition, structural images were acquired in the same scanning session. Here, a T1-weighted magnetization prepared rapid gradient echo sequence was used (TE 4.2 ms; TR 2300 ms; spatial resolution, 1.1 × 1 × 1 mm; scan time, 7.45 min). A clinical neuroscientist with extensive neuroradiological experience checked the T1-weighted images for lesions and structural malformations to ensure that brain scans were void of morphological abnormalities.

### Hypothalamus-specific DWI preprocessing

Image processing was carried out with default parameters unless specified otherwise. After correction for eddy currents, head movement, and removal of non-brain tissue, principal eigenvalues *λ*1, *λ*2, and *λ*3 (weighted least squares) and the derived diffusion scalar MD (=mean of principal eigenvalues) were calculated using the FMRIB software library (v5.0.6; http://fsl.fmrib.ox.ac.uk/fsl/fslwiki/). To localize the hypothalamus with high spatial specificity, a binary mask was created including brainstem and midbrain (*x*/*y*/*z* = 30…−30/5…−60/5…−50 mm MNI-space) (Hahn et al. 2013). Subsequent spatial normalization (affine transformations only with averaged size template regularization) was optimized using the binary mask as weighting image for the cost function in SPM8 (http://www.fil.ion.ucl.ac.uk/spm/software/spm8/). The spatial transformation matrix was estimated with the T1-weighted image. To provide optimal image alignment between the three DTI measurements, MD images were first realigned in a two-pass procedure in SPM8 (quality = 1, register to mean). The average image was then coregistered to the T1-weighted image and transformation matrices of coregistration and normalization were then applied to each of the 3 MD images. Normalized MD images were then again realigned as previously done for longitudinal analyses (Lanzenberger et al. 2013). Reslicing was set to 1 × 1 × 1 mm and data were smoothed with a 4 mm Gaussian kernel. Such optimized procedures have been demonstrated to yield a marked improvement on deep brain structure registration accuracy (Eippert et al. 2009; Napadow et al. 2006).

### Statistical analysis

Group statistics were calculated with random-effects models in SPM8. First, repeated-measures ANOVA using time as within-subjects factor (MRI 1, MRI 2, and MRI 3) and group (FC, FtM, and MC) as between-subjects factor was performed. Post hoc separate models and pairwise comparisons were conducted to investigate group differences and MD changes over time for each group separately. Finally, MD changes over time were associated with changes in hormone plasma levels (ordered by rank) and individual MD variability at baseline was associated with hormone plasma levels at baseline using regression analysis. All statistical tests were evaluated within an a priori defined region around the hypothalamus with small volume correction (*x*/*y*/*z* = 19…−19/5…−19/3…−20 mm). All tests are presented at *p* ≤ 0.05 family wise error (FWE) correction at voxel-level unless specified otherwise.

## Results

### Hormone treatment

Hormone levels as well as hormone-level changes over time were not normally distributed (*p* < 0.05, Kolmogorov–Smirnov test). As expected, testosterone plasma levels significantly increased in FtMs due to androgen treatment (*p* < 0.001, Friedman two-way ANOVA by ranks). Similarly, bioavailable testosterone and the free androgen index significantly increased, whereas SHBG levels significantly decreased over time (*p* < 0.001; for quartiles and pairwise comparisons, see Table 1). No significant changes were observed for estradiol in FtMs and no significant mean hormone-level changes were observed in the control groups (*F* test).

**Table 1:** Plasma hormone levels before (MRI 1), after 1 month (MRI 2) and after 4 months (MRI 3) of testosterone treatment in FtMs and hormone-level fluctuations in FCs and MCs

	FC			FtM			MC		
	MRI 1	MRI 2	MRI 3	MRI 1	MRI 2	MRI 3	MRI 1	MRI 2	MRI 3
*T*_ng/ml_	0.30	0.17	0.18	0.23	2.23	3.02	4.28	3.87	3.87
	**0.38**	**0.31**	0.28*	**0.36**	3.56**	3.84**	**5.75**	**6.03**	**5.30**
	0.43	0.45	0.38	0.44	5.36	4.92	6.65	7.16	7.44
Bioav *T*_ng/ml_	0.07	0.05	0.04	0.06	0.85	1.52	2.34	1.99	1.77
	**0.09**	**0.13**	**0.06**	**0.10**	1.50**	1.93**	**2.73**	**2.76**	**2.40**
	0.15	0.15	1.00	0.17	2.17	2.22	2.88	3.36	3.31
FAI	0.36	0.18	0.24	0.35	5.37	10.50	13.41	12.50	11.62
	**0.54**	**0.39**	**0.36**	**0.57**	9.29**	**12.79** ^**,#^	**15.87**	**16.28**	**15.07**
	1.00	0.88	0.80	1.01	16.36	16.15	18.76	18.05	18.00
*E*_2 pg/ml_	30.25	45.75	35.25	55.50	26.50	31.00	17.00	15.50	13.75
	**65.00**	**67.50**	**81.00**	**114.00**	**43.00**	**45.00**	**21.00**	**19.00**	**22.50**
	240.25	245.50	128.25	145.00	112.50	104.00	27.50	34.00	30.75
SHBG	36.88	30.50	36.73	42.70	27.95	25.40	27.28	27.65	31.03
	**56.40**	**50.20**	**62.10**	**66.10**	37.20**	**30.90** ^**,#^	**37.90**	**38.50**	**40.50**
	103.55	90.60	104.28	83.80	50.95	38.65	46.80	49.70	47.13

Depicted are percentiles 25, 50 (median), and 75

*FAI* free androgen index

Bold indicates 50 (median) percentile values

* Significant difference from MRI 1 at *p* < 0.05, **p < 0.01, and ***p < 0.001

^#^Significant difference from MRI 2 at *p* < 0.05

### Hypothalamic microstructure changes

**Fig. 1:**
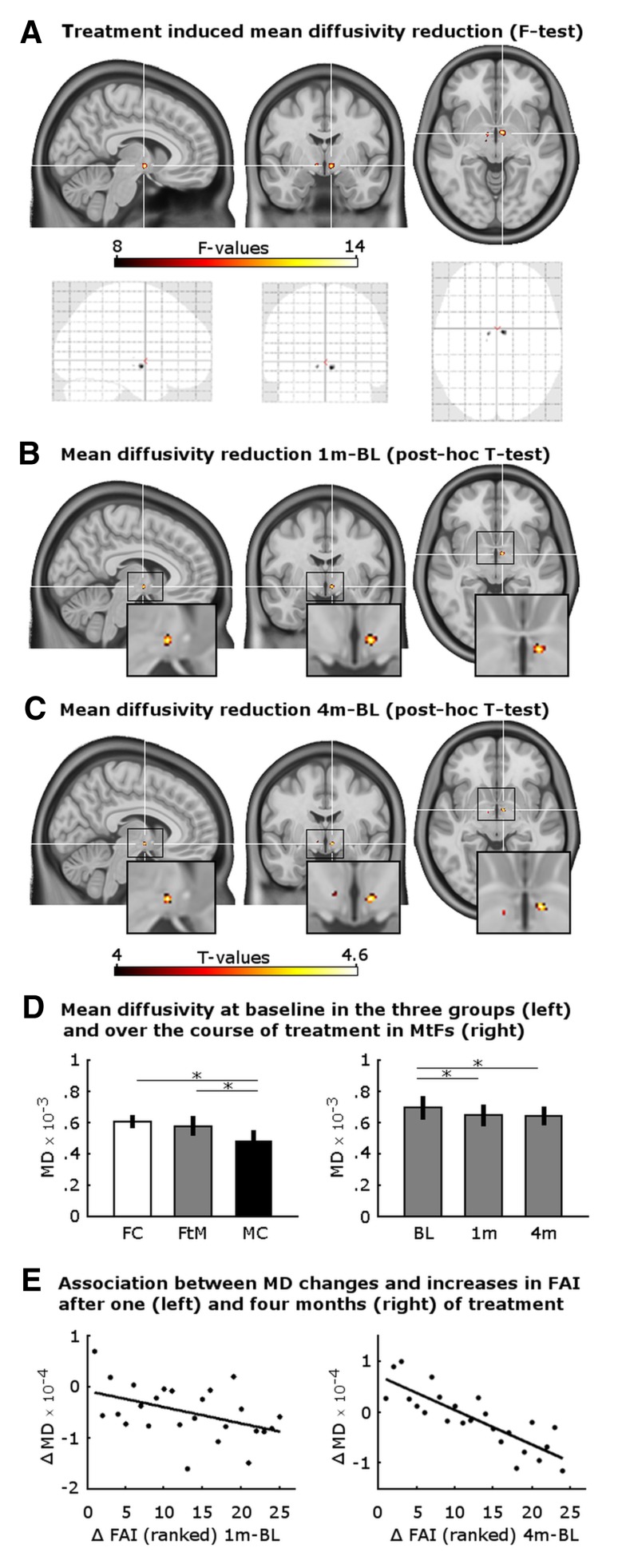
Effects of testosterone treatment on mean diffusivity (MD) in the lateral hypothalamus in female-to-male transgender persons. **a** Significant MD changes over the course of treatment (*F* test of the repeated-measures ANOVA) in the right and left lateral hypothalamus, crosshair at *x*/*y*/*z* = 7/−4/−6 mm MNI-space. **b** Significant MD reduction after 1 month of treatment (crosshair is at *x*/*y*/*z* = 7/−4/−6 mm MNI-space) and **c** after 4 months of treatment (crosshair is at *x*/*y*/*z* = 7/−4/−7 mm MNI-space). Displayed are significant voxels at a threshold of *p* < 0.0001 uncorrected. **d** *Left* bar chart depicts MD values (mm^2^/s, mean ± SD) at baseline for *x*/*y*/*z* = −9/−9/−6 mm MNI-space, *FC* female controls, *FtM* female to males, *MC* male controls; *right* bar chart depicts MD values in FtMs at baseline, after 1 month, and after 4 months of testosterone treatment (BL, 1, 4 m, respectively) for *x*/*y*/*z* = 7/−4/−6 mm MNI-space. **e** Scatter plots depict associations between changes in free androgen index and changes in hypothalamic MD values after 1 month of treatment in FtMs in the right lateral hypothalamus at *x*/*y*/*z* = 7/−4/−6 mm MNI-space (*left*) and after 4 months of treatment in the left lateral hypothalamus at *x*/*y*/*z* = −7/−1/−10 mm MNI-space (*right*)

Repeated-measures ANOVA revealed a main effect of group in the left hypothalamus (*F* = 109.69, *p* < 0.001, peak voxel at −1/0/−5 mm) but no main effect of time and no interaction effect that survived correction for multiple comparisons. At an uncorrected significance level, a main effect of time (*F* = 10.62, *p* < 0.001 at 9/−3/−7) and an interaction between group and time (*F* = 5.46, *p* = 0.001 at −9/−7/−6) were observed. A separate model only including FtMs revealed a significant change in MD values over the course of testosterone treatment in the right and left lateral hypothalamus (*F* = 14.76, *p* = 0.035, peak voxel at 7/−4/−6 mm, see Fig. 1a, d). Post hoc comparisons indicated a significant MD reduction after 1 month of treatment in the same cluster (*T* = −4.77, *p* = 0.024, see Fig. 1b) and after 4 months of treatment bilaterally in right and left hypothalamus when compared to the baseline measurement before treatment (*T* = −4.65, and *T* = −4.68, *p* < 0.05, at 7/−4/−7 and −9/−6/−6 mm, respectively, see Fig. 1c). Including treatment regimen as factor did not change the significance or anatomical location of these findings. No significant MD increases were observed in FtMs and no significant MD changes over time (neither *F* test nor post hoc comparisons) were observed in the two control groups.

Comparing all three groups at baseline revealed significant group differences in MD values within the left hypothalamus (*F* = 16.77, *p* = 0.008, peak voxel at −8/−8/−6 mm, see Fig. 1d). Post hoc comparisons indicated significantly lower MD values for MCs compared to FtMs in the left (*T* = −4.50, *p* = 0.03, −9/−9/−6 mm) and compared to FCs in the left and right hemispheres (*T* = −5.67, *p* = 0.001 and *T* = −4.74, *p* = 0.015, −8/−8/−6 and 12/−14/−8 mm, respectively). Interestingly, group differences at baseline became non-significant after including testosterone plasma levels as nuisance variable in the analysis. This indicates that testosterone mediates observed sex differences on MD values at baseline. Taken together, our results show that FtMs had female MD values at baseline, which decreased towards male levels after testosterone treatment.

### Associations between hormones and hypothalamic microstructure

Next, associations between MD reductions and increases in testosterone in FtMs were investigated using regression analysis. Increases in free androgen index (FAI) significantly predicted MD reductions after 1 month of treatment (*T* = −2.41, *p* = 0.024, uncorrected, see Fig. 1e) in the peak voxel with strongest MD reduction in the ANOVA (7/−4/−6 mm, see above). Similarly, bioavailable testosterone predicted MD reductions with borderline significance (*T* = −2.02, *p* = 0.056, uncorrected), whereas no significance was present for total testosterone (*p* = 0.25) and no significant predictions were observed for changes after 4 months of treatment. To reduce the likelihood of a spurious finding, voxel-wise regression was performed for the entire hypothalamic volume. The analysis confirmed associations between FAI/bioavailable testosterone increases and MD reductions in the right and left hypothalamus at an uncorrected level of *p* < 0.001 in several clusters (FAI: *T* = −3.84, 6/0/−3, *T* = 3.84, −3/−6/−12, *T* = 3.75, 7/4/−5; bioavailable testosterone: *T* = 4.00, 7/4/−5). Instead, significant associations corrected for multiple comparisons were observed for changes after 4 months of treatment in the left hypothalamus (FAI: *T* = −7.06, *p* = 0.002, at −7/−1/−10, see Fig. 1e; bioavailable testosterone: *T* = −6.68, *p* = 0.005, at −6/1/−8; total testosterone: *T* = −6.78, *p* = 0.003, at −5/5/−7). Regression analysis between voxel-wise MD reductions and estradiol level changes revealed positive associations (*T* = 3.55, *p* = 0.001 at −4/−6/−11 and *T* = 3.63, *p* = 0.001 at 4/−6/−11) although only at an uncorrected significance level.

Finally, we examined whether individual testosterone levels are associated with MD values at baseline. This cross-sectional association revealed no significant effect for either group or hormone, although at uncorrected levels, a negative effect of bioavailable testosterone was present in the left ventral hypothalamus (*T* = −3.79, *p* < 0.001, uncorrected at −2/−2/−20).

## Discussion

Here, we show MD reductions in the hypothalamus from female towards male proportions in FtMs undergoing hormone replacement therapy with testosterone. Regression analysis further indicates that increases in free androgen index and in bioavailable testosterone plasma levels are associated with the observed MD reduction in the hypothalamus. The adult hypothalamus has a volume of approximately 0.7 cm^3^ per hemisphere and its subparts can be reliably measured using MRI, even with machines at the lower field strength such as 1.5 T (Lemaire et al. 2013). Hypothalamic nuclei are well described and anatomical landmarks indicate MD reductions in our study located in the lateral hypothalamic area (Lemaire et al. 2013; Baroncini et al. 2012).

Using diffusion-weighted MRI, the previous studies found activity-related diffusion changes in hypothalamic substructures that are likely generated by astrocytic volume changes (Lizarbe et al. 2013). As hypothalamic activity is strongly modulated by gonadal steroids (Campbell and Herbison 2014), we speculate that the reductions in MD in the lateral hypothalamus in our study were caused by testosterone treatment. Thus, MD reductions after testosterone treatment may indicate increased activity and associated cellular swelling in the lateral hypothalamus (Lizarbe et al. 2013; Le Bihan and Iima 2015). Indeed, early animal research shows significant increases in the firing rate of lateral hypothalamic neurons in response to injections of testosterone (Orsini 1982). Lesion and stimulation studies have associated the lateral hypothalamus with motivation and reward (Stuber and Wise 2016). This is supported by studies elucidating reward-related connections to the dopaminergic ventral tegmental area (Shizgal et al. 1980). Moreover, orexin-producing neurons are known to regulate arousal, as well as feeding and other reward-related behaviors and are restricted to the lateral hypothalamus (Rosin et al. 2003). In particular, effects of testosterone on lateral hypothalamus neurons have been linked to male sexual motivation (Hendricks and Scheetz 1973). This coincides well with the observation that sexual desires are fundamentally increased after initiation of testosterone treatment in FtMs (Wierckx et al. 2011) and that frequency of sexual intercourse is positively correlated with testosterone levels (Costantino et al. 2013). Hence, presumed increases in lateral hypothalamic activity and associated MD reductions may be linked to increased sexual arousal in FtMs. Unfortunately, we were not able to directly test this as sexual arousal was not systematically assessed in our study.

Precaution may also be put forward when it comes to the consistency of anatomical locations of results. Whereas MD reduction was most prominent in the right lateral hypothalamus and only bilaterally significant after 4 months of treatment, testosterone plasma-level increases predicted MD reductions predominantly in the left lateral hypothalamus. In any case, given the imprecision in spatial specificity introduced by motion in small structures such as the hypothalamus, our results might suggest a more general testosterone effect on lateral hypothalamus rather than an anatomically specific and asymmetric influence. Moreover, the action of testosterone on hypothalamic tissue is complex and implies several cellular events. This includes early responses within seconds that can be explained by non-genomic effects as well as late responses, which are compatible with the general model of steroid-hormone action (McEwen et al. 2015). Finally, it is generally assumed that testosterone exerts its influence on male sexual behavior through transformation into estradiol, although see (Antonio-Cabrera and Paredes 2014). Whether this “aromatization hypothesis” also applies for MD changes observed in our study remains to be investigated.

Of note, an earlier study by Hulshoff-Pol et al. (2006) investigated the effects of sex hormone replacement therapy on hypothalamic volume in 6 FtMs and 8 MtFs. They observed that 4 months of androgen treatment in FtMs increased hypothalamus volume and decreased lateral and third ventricle volumes, whereas anti-androgen and estrogen therapy in MtFs had the opposite effect. Although interesting and promising, we refrained from performing an additional volumetric analysis of our data given the significant challenges and shortcomings of MRI investigations on hypothalamus volume in vivo (Schindler et al. 2012). First, ventricular dilation and volume changes of surrounding structures may affect the reliability of hypothalamus volume measurements (Hulshoff-Pol et al. 2006). Second, manual delineation criteria for the hypothalamus region of interest (ROI) differ between studies and are influenced by the delineator. Third, there is strong inter-individual intensity variation of hypothalamus boundaries which even compromise advantages of high-resolution imaging such as at 7T (Schindler et al. 2017). Finally, visible landmarks in MRI data may not coincide with histology which questions the validity of volumetric analyses.

Several limitations of this study should be noted. First, it is important to note that the interaction between group and time did not withstand correction for multiple comparisons. Thus, we cannot conclude that groups were significantly different in their changes of MD over time (Nieuwenhuis et al. 2011). We base our interpretation on the fact that only the group receiving intervention showed MD changes, while the others did not, and because testosterone level changes, i.e., the “size of intervention” correlated with changes in MD. Second, besides testosterone, several other variables—related or not related to hormone replacement therapy—may in principle explain our finding. However, based on our quasi-experimental approach, we believe that it is justified to argue that testosterone treatment or at least some variable associated with the intervention is the most parsimonious explanation for observed effects in the FtM group. This is not to say that there are various possibilities of mediators or moderators (e.g., other hormones) between testosterone treatment and MD changes. In that respect, it is noteworthy that estradiol plasma-level variability was positively correlated with MD changes at an uncorrected *p* value level, although estradiol plasma levels did not change significantly over the course of treatment. Third, we did not test for behavioral changes associated with our finding. This includes not only sexual arousal as stated earlier but may also apply to changes in mood, mindsets, or habits. Finally, hypothalamic diffusion parameters may vary between fasted and fed states (Lizarbe et al. 2013). Yet, satiety was not investigated in our study which potentially increased variability in our dependent variable.

Together, our data indicate that testosterone treatment reduces mean diffusivity in the adult lateral hypothalamus and that treatment induced increases in testosterone levels are associated with the magnitude of MD reductions. These findings imply microstructural changes and may indicate related changes in neural activity by testosterone in the human hypothalamus of female-to-male transgender persons towards male levels.

## References

[CR1] Ahmed EI, Zehr JL, Schulz KM, Lorenz BH, DonCarlos LL, Sisk CL (2008). Pubertal hormones modulate the addition of new cells to sexually dimorphic brain regions. Nat Neurosci.

[CR2] Alkan A, Sahin I, Keskin L, Cikim AS, Karakas HM, Sigirci A, Erdem G (2008). Diffusion-weighted imaging features of brain in obesity. Magn Reson Imaging.

[CR3] Antonio-Cabrera E, Paredes RG (2014). Testosterone or oestradiol implants in the medial preoptic area induce mating in noncopulating male rats. J Neuroendocrinol.

[CR4] Arnold AP (2009). The organizational-activational hypothesis as the foundation for a unified theory of sexual differentiation of all mammalian tissues. Horm Behav.

[CR5] Baroncini M, Jissendi P, Catteau-Jonard S, Dewailly D, Pruvo JP, Francke JP, Prevot V (2010). Sex steroid hormones-related structural plasticity in the human hypothalamus. Neuroimage.

[CR6] Baroncini M, Jissendi P, Balland E, Besson P, Pruvo JP, Francke JP, Dewailly D, Blond S, Prevot V (2012). MRI atlas of the human hypothalamus. Neuroimage.

[CR7] Campbell RE, Herbison AE (2014). Gonadal steroid neuromodulation of developing and mature hypothalamic neuronal networks. Curr Opin Neurobiol.

[CR8] Costantino A, Cerpolini S, Alvisi S, Morselli PG, Venturoli S, Meriggiola MC (2013). A prospective study on sexual function and mood in female-to-male transsexuals during testosterone administration and after sex reassignment surgery. J Sex Marital Ther.

[CR9] Eippert F, Bingel U, Schoell ED, Yacubian J, Klinger R, Lorenz J, Buchel C (2009). Activation of the opioidergic descending pain control system underlies placebo analgesia. Neuron.

[CR10] Hahn A, Kranz GS, Seidel EM, Sladky R, Kraus C, Kublbock M, Pfabigan DM, Hummer A, Grahl A, Ganger S, Windischberger C, Lamm C, Lanzenberger R (2013). Comparing neural response to painful electrical stimulation with functional MRI at 3 and 7 T. Neuroimage.

[CR11] Hahn A, Kranz GS, Sladky R, Kaufmann U, Ganger S, Hummer A, Seiger R, Spies M, Vanicek T, Winkler D, Kasper S, Windischberger C, Swaab DF, Lanzenberger R (2016). Testosterone affects language areas of the adult human brain human brain mapping early view. Hum Brain Map.

[CR12] Hendricks SE, Scheetz HA (1973). Interaction of hypothalamic structures in the mediation of male sexual behavior. Physiol Behav.

[CR13] Hulshoff-Pol H, Cohen-Kettenis P, Van Haren N, Peper J, Brans R, Cahn W, Schnack H, Gooren L, Kahn R (2006). Changing your sex changes your brain: influences of testosterone and estrogen on adult human brain structure. Eur J Endocrinol.

[CR14] Kenealy BP, Kapoor A, Guerriero KA, Keen KL, Garcia JP, Kurian JR, Ziegler TE, Terasawa E (2013). Neuroestradiol in the hypothalamus contributes to the regulation of gonadotropin releasing hormone release. J Neurosci.

[CR15] Kranz GS, Hahn A, Kaufmann U, Kublbock M, Hummer A, Ganger S, Seiger R, Winkler D, Swaab DF, Windischberger C, Kasper S, Lanzenberger R (2014). White matter microstructure in transsexuals and controls investigated by diffusion tensor imaging. J Neurosci.

[CR16] Lanzenberger R, Baldinger P, Hahn A, Ungersboeck J, Mitterhauser M, Winkler D, Micskei Z, Stein P, Karanikas G, Wadsak W, Kasper S, Frey R (2013). Global decrease of serotonin-1A receptor binding after electroconvulsive therapy in major depression measured by PET. Mol Psychiatry.

[CR17] Le Bihan D, Iima M (2015). Diffusion magnetic resonance imaging: what water tells us about biological tissues. PLoS Biol.

[CR18] Le Bihan D, Urayama S, Aso T, Hanakawa T, Fukuyama H (2006). Direct and fast detection of neuronal activation in the human brain with diffusion MRI. Proc Natl Acad Sci USA.

[CR19] Lemaire JJ, Nezzar H, Sakka L, Boirie Y, Fontaine D, Coste A, Coll G, Sontheimer A, Sarret C, Gabrillargues J, De Salles A (2013). Maps of the adult human hypothalamus. Surg Neurol Int.

[CR20] Levy A, Crown A, Reid R (2003). Endocrine intervention for transsexuals. Clin Endocrinol (Oxf).

[CR21] Lizarbe B, Benitez A, Sanchez-Montanes M, Lago-Fernandez LF, Garcia-Martin ML, Lopez-Larrubia P, Cerdan S (2013). Imaging hypothalamic activity using diffusion weighted magnetic resonance imaging in the mouse and human brain. Neuroimage.

[CR22] Madeira MD, Ferreira-Silva L, Paula-Barbosa MM (2001). Influence of sex and estrus cycle on the sexual dimorphisms of the hypothalamic ventromedial nucleus: stereological evaluation and Golgi study. J Comp Neurol.

[CR23] McCarthy MM, Wright CL, Schwarz JM (2009). New tricks by an old dogma: mechanisms of the Organizational/Activational Hypothesis of steroid-mediated sexual differentiation of brain and behavior. Horm Behav.

[CR24] McEwen BS, Gray JD, Nasca C (2015). 60 years of neuroendocrinology: redefining neuroendocrinology: stress, sex and cognitive and emotional regulation. J Endocrinol.

[CR25] Mizuno M, Terasawa E (2005). Search for neural substrates mediating inhibitory effects of oestrogen on pulsatile luteinising hormone-releasing hormone release in vivo in ovariectomized female rhesus monkeys (*Macaca mulatta*). J Neuroendocrinol.

[CR26] Napadow V, Dhond R, Kennedy D, Hui KK, Makris N (2006). Automated brainstem co-registration (ABC) for MRI. Neuroimage.

[CR27] Nieuwenhuis S, Forstmann BU, Wagenmakers EJ (2011). Erroneous analyses of interactions in neuroscience: a problem of significance. Nat Neurosci.

[CR28] Orsini JC (1982). Androgen influence on lateral hypothalamus in the male rat: possible behavioral significance. Physiol Behav.

[CR29] Rosin DL, Weston MC, Sevigny CP, Stornetta RL, Guyenet PG (2003). Hypothalamic orexin (hypocretin) neurons express vesicular glutamate transporters VGLUT1 or VGLUT2. J Comp Neurol.

[CR30] Sagi Y, Tavor I, Hofstetter S, Tzur-Moryosef S, Blumenfeld-Katzir T, Assaf Y (2012). Learning in the fast lane: new insights into neuroplasticity. Neuron.

[CR31] Schindler S, Geyer S, Strauss M, Anwander A, Hegerl U, Turner R, Schonknecht P (2012). Structural studies of the hypothalamus and its nuclei in mood disorders. Psychiatry Res.

[CR32] Schindler S, Schreiber J, Bazin PL, Trampel R, Anwander A, Geyer S, Schonknecht P (2017). Intensity standardisation of 7T MR images for intensity-based segmentation of the human hypothalamus. PLoS One.

[CR33] Shizgal P, Bielajew C, Corbett D, Skelton R, Yeomans J (1980). Behavioral methods for inferring anatomical linkage between rewarding brain stimulation sites. J Comp Physiol Psychol.

[CR34] Silva NL, Boulant JA (1986). Effects of testosterone, estradiol, and temperature on neurons in preoptic tissue slices. Am J Physiol.

[CR35] Spies M, Hahn A, Kranz GS, Sladky R, Kaufmann U, Hummer A, Ganger S, Kraus C, Winkler D, Seiger R, Comasco E, Windischberger C, Kasper S, Lanzenberger R (2016). Gender transition affects neural correlates of empathy: a resting state functional connectivity study with ultra high-field 7T MR imaging. Neuroimage.

[CR36] Stuber GD, Wise RA (2016). Lateral hypothalamic circuits for feeding and reward. Nat Neurosci.

[CR37] Tu CH, Niddam DM, Yeh TC, Lirng JF, Cheng CM, Chou CC, Chao HT, Hsieh JC (2013). Menstrual pain is associated with rapid structural alterations in the brain. Pain.

[CR38] Wierckx K, Elaut E, Van Caenegem E, Van De Peer F, Dedecker D, Van Houdenhove E, T’Sjoen G (2011). Sexual desire in female-to-male transsexual persons: exploration of the role of testosterone administration. Eur J Endocrinol.

[CR39] Xu Y, Nedungadi TP, Zhu L, Sobhani N, Irani BG, Davis KE, Zhang X, Zou F, Gent LM, Hahner LD, Khan SA, Elias CF, Elmquist JK, Clegg DJ (2011). Distinct hypothalamic neurons mediate estrogenic effects on energy homeostasis and reproduction. Cell Metab.

